# Biocompatibility and Favorable Response of Mesenchymal Stem Cells on Fibronectin-Gold Nanocomposites

**DOI:** 10.1371/journal.pone.0065738

**Published:** 2013-06-24

**Authors:** Huey-Shan Hung, Cheng-Ming Tang, Chien-Hsun Lin, Shinn-Zong Lin, Mei-Yun Chu, Wei-Shen Sun, Wei-Chien Kao, Hsieh Hsien-Hsu, Chih-Yang Huang, Shan-hui Hsu

**Affiliations:** 1 Graduate Institute of Basic Medical Science, China Medical University, Taichung, Taiwan, R. O. C.; 2 Center for Neuropsychiatry, China Medical University Hospital, Taichung, Taiwan, R.O.C.; 3 Institute of Oral Sciences, Chung Shan Medical University, Taichung, Taiwan, R.O.C.; 4 China Medical University Beigang Hospital, Yunlin, Taiwan, R.O.C.; 5 Graduate Institute of Immunology, China Medical University, Taichung, Taiwan, R.O.C.; 6 Blood Bank, Taichung Veterans General Hospital, Taichung, Taiwan, R.O.C.; 7 School of Chinese Medicine, China Medical University, Taichung, Taiwan, R.O.C.; 8 Institute of Polymer Science and Engineering, National Taiwan University, Taipei, Taiwan, R.O.C.; 9 Rehabilitation Engineering Research Center, National Taiwan University, Taipei, Taiwan, R.O.C.; University of California, San Diego, United States of America

## Abstract

A simple surface modification method, comprising of a thin coating with gold nanoparticles (AuNPs) and fibronectin (FN), was developed to improve the biocompatibility required for cardiovascular devices. The nanocomposites from FN and AuNPs (FN-Au) were characterized by the atomic force microscopy (AFM), UV-Vis spectrophotometry (UV-Vis), and Fourier transform infrared spectroscopy (FTIR). The biocompatibility of the nanocomposites was evaluated by the response of monocytes and platelets to the material surface in vitro. FN-Au coated surfaces demonstrated low monocyte activation and platelet activation. The behavior of human umbilical cord-derived mesenchymal stem cells (MSCs) on FN-Au was further investigated. MSCs on FN-Au nanocomposites particularly that containing 43.5 ppm of AuNPs (FN-Au 43.5 ppm) showed cell proliferation, low ROS generation, as well as increases in the protein expression levels of matrix metalloproteinase-9 (MMP-9) and endothelial nitric oxide synthase (eNOS), which may account for the enhanced MSC migration on the nanocomposites. These results suggest that the FN-Au nanocomposite thin film coating may serve as a potential and simple solution for the surface modification of blood-contacting devices such as vascular grafts.

## Introduction

Surface modification of biomaterials by immobilization of different biomolecules has been proven to improve blood compatibility [1] or to enhance cell attachment and proliferation [2]. Fibronectin (FN) is a well studied glycoprotein in the extracellular matrix (ECM). It is widely distributed in the connective tissue and blood plasma of human body [3]. FN also serves to organize cellular interaction with ECM by binding to different components of ECM and to membrane-bound FN receptors on cell surfaces [4]. ECM presents an abundance of macromolecules with sizes featured at the nanometer scale. The influence of surface topography on the adhesion and differentiation of osteoblast-like cells was enhanced by the surface adsorbed FN [5]. FN immobilized on silanized Ti surface was found to enhance the attachment of fibroblasts [6]. Besides, plasma FN and fibrinogen play an important role in establishing the provisional matrix after the inflammatory phase [7]. This implicates FN in ECM as a key molecule in cardiovascular pathophysiology.

Gold (Au) is one of the noble metals with high biocompatibility. Au nanoparticles (AuNPs) were used for immobilization of biomolecules such as proteins, enzymes, and antibodies [8]. When embedded at a proper amount in a synthetic polymer such as polyurethane, AuNPs may alter the surface morphology of the polymer and prevent it from causing blood clotting [9]–[14].

Stem cell homing and migration are critical processes for the ongoing replacement of mature cells and regeneration of damaged cells in many adult tissues [15]. Mesenchymal stem cell (MSC) mobilization from bone marrow enables their migration to peripheral blood and homing to peripheral tissues. This process is tightly controlled by specialized signals [16] and requires interplay of adhesion molecules, cytokines and chemokines, and ECM degrading proteases [17], [18]. Activated endothelial cells (ECs) express the dimeric transmembrane αVβ3 integrin, which interacts with ECM proteins (vitronectin and fibronectin) and regulates the migration of ECs through ECM during vessel formation [19]. The activated ECs synthesize proteolytic enzymes, such as matrix metalloproteinases (MMPs), to degrade the basement membrane and ECM [20]. Our previous study showed that stem cell homing was linked with activation of CXCR4, Rho GTPase, and the focal adhesion kinase (FAK), subsequently resulting in MMP activity and cell migration [21]. Embedding AuNPs in polyurethane was found to trigger EC migration by phosphatidylinositol 3-kinase (PI3K)/Akt/endothelial nitric oxide synthase (eNOS) activation and FAK signaling [11], [13], [22]. Polyurethane, however, is an artificial substance that can result in foreign body reactions.

Different forms of nanotopography, including nanograting, nanopost, and nanopit, have been fabricated for investigation of the cellular response. The nanoscaled features presented by nanotopography can lead to changes in the number, size, and arrangement of focal adhesions signaling and alter cellular behavior, such as migration and differentiation [23], [24]. Investigators have also utilized nanotopography to direct stem cell differentiation, such as the osteoblastic and neuronal differentiation of mesenchymal stem cells and embryonic stem cells [25]–[28]. Although nanotopography was found to induce changes in focal adhesion, cytoskeletal organization, and mechanical properties of human mesenchymal stem cells [29], the exact mechanisms by which nanotopography influences the behavior in different types of stem cells remain unclear.

Since FN is readily adsorbed on a wide variety of material surfaces, the surface modification by FN may be achieved by simple coating. In this study, we investigated if the combination of FN and AuNPs may produce anti-inflammatory and anti-platelet effects and may induce the migration and EC phenotype of human MSCs. This would help determine if FN-Au nanocomposites could serve as a convenient coating for surface modification of blood-contacting devices.

## Materials and Methods

### 1. Preparation of fibronectin-gold (FN-Au) nanocomposite coatings

Human fibronectin was purchased from Millipore Corporation (USA). The solution of gold nanoparticles (AuNPs) was supported by Gold Nanotech Inc. (Taiwan). Gold nanoparticle was dispersion into distilled water (∼17.5 ppm). The diameter of the AuNPs was ∼5 nm [15]. FN-Au solutions were prepared by mixing FN solution (1 mg/ml) with a certain amount of AuNPs (17.4, 43.5, and 174 ppm of Au in the final dry weight). The amount of FN coated to culture dish by applying the total surface area (cm^2^) by the desired concentration amount is 40 µg/cm^2^ of the solution was covered on culture dish, culture plate or 15 mm round coverslip glass. The solution was allowed to adsorb to the surface of the culture area for 20–30 min. Removed residual mixture solution before proceeding standard cell culture procedures to obtain the coating of FN or the FN-Au nanocomposites (denoted “FN-Au”).

### 2. Surface characterization of FN and the FN-Au nanocomposites

The UV/Vis spectra of FN and FN-Au solutions were obtained by a UV/Vis spectrophotometer (Helios Zeta, Thermo Fisher, USA). The surface morphology of the dry coating was examined by an atomic force microscope (AFM) equipped with a 100-µm piezoelectric scanner (JEOL JSM-6700 F, Japan). The images were obtained in the tapping mode in air with a triangular cantilever (force constant of 21–78 N/m) supporting an integrated pyramidal tip of Si_3_N_4_. The average roughness of the surface was analyzed using Image Pro Plus 4.5 software. The surface morphology in wet conditions (was performed using a molecular imaging system (PicoTREC™ AFM, Molecular Imaging, USA) operating in tapping mode. Images were recorded in culture medium (without serum). Symmetric tip sharpened ContAl-G cantilevers (E-V Nanotech, Taiwan with a resonance frequency of 13 kHz in aqueous medium) were used. Surface areas of 500×500 nm were evaluated using the PicoPlus™ system program and the images were analyzed by the Gwyddion image processing software (version 2.31, Czech Metrology Institute, Czech. In addition, the coating was chemically analyzed using an FTIR (Shimadzu FTIR Model IR Pretige-21, Japan). Each sample was scanned eight times in the spectral region of 400–4000 cm^−1^ with a resolution setting of 2 cm^−1^ and averaged to produce each spectrum.

### 3. Biocompatibility assays

#### 3.1 Monocyte activation test

Monocytes were isolated from human blood sample of volunteer donors by density gradient centrifugation using Percoll (Sigma, USA). Cells were suspended in a medium of RPMI containing 10% FBS and 1% (v/v) antibiotic (10,000 U/ml penicillin G and 10 mg/ml streptomycin) and the cell concentration was adjusted to 1×105 cells/ml. FN and FN-Au were coated on 24-well tissue culture plates. One milliliter of the cell suspension was added to each well and allowed to adhere for 96 h at 37°C in 5% CO2. The adherent cells were trypsinized and the numbers of monocytes and macrophages were counted based on their morphology by a hemocytometer combined with the inverted phase contrast microscope. The ratio between numbers of monocytes and macrophages (i.e. the conversion ratio) was used as an inflammatory index [10], [30].

#### 3.2 Platelet activation test

FN and FN-Au were coating on coverslip glass and were placed in a 24-well culture plate and 0.5 ml of platelet-rich plasma (∼2×106 platelets/m), which were obtained from the Taichung Veterans General Hospital, Taichung, Taiwan). Samples were removed after incubation for 1 h and the number of adherent platelets were counted by a cell counter (Assistant, Germany). For platelet activation, samples were fixed by 2.5% glutaraldehyde (Sigma, USA)/phosphate buffered saline (PBS) (pH = 7.4), dehydrated in ethanol solutions (increasing concentration from 30% to 100%), critical point-dried, sputter-coated with gold, and examined by a scanning electron microscope (SEM) (JEOL JSM-6700 F, Japan). Each platelet was identified for the morphological change and the degree of activation was scored by: 0 = round (in unactivated type), 1/4 = dendritic (in pseudopodial but no flattening type), 1/2 = spread-dendritic (in flattened pseudopodia type), 3/4 = spreading (in late phase pseudopodial with hyaloplasm spreading type), and 1 = fully spread (in totally activated type). The average degree (mean score) of platelet activation on different materials was quantified based on 50 adherent platelets determined by SEM [10].

### 4. Behaviors of MSCs on FN and FN-Au

#### 4.1 Cell morphology by fluorescence labeling of cytoskeletal fibers and by SEM

Human umbilical cord (Wharton's jelly)-derived mesenchymal stem cells (MSCs) were kindly provided by Prof. Woei-Cherng Shyu [31] and maintained in condition medium [high glucose Dulbecco's Modified Eagle's Medium (DMEM) (Invitrogen), supplemented with 1% (v/v) antibiotics 100 U/ml penicillin/streptomycin, 1% sodium pyruvate]. Cells of passages 8–20 were used in this study. The genuineness of MSCs was confirmed by flow cytometry (**Figure S1**). MSCs were seeded in 24-well plates with FN or FN-Au coated glass coverslips at a density of 0.5×10^4^ cells per well after 8 h and 48 h of incubation. Cells cultured in a blank well (coverslip glass) were used as a control. For cytoskeletal examination, cells were fixed with 4% paraformaldehyde/with PBS buffer for 15 min and permeabilized with 0.5% (v/v) Triton-X 100 (Sigma, USA) in PBS buffer for 10 min prior to staining. After then, 5% (v/v) of bovine serum albumin (BSA) (Sigma, USA) was used to block the non-specific binding. Finally, cells were stained with appropriate concentration of rhodamine phalloidin (∼6 µM) (Sigma, USA) for 30 min. Cell nuclei were co-stained with 4′, 6-diamidino-2-phenylindole (DAPI) (∼1 µg/ml). On the other hand, samples for SEM assay were fixed by the standard procedure previously described.

#### 4.2 Cell proliferation test

FN and FN-Au were coated into the bottom of 96-well tissue culture plates. Two hundred µl of MSC suspension with a density of 6×10^3^ cells/ml was injected into each well of the culture plates. Cells cultured in a blank well (tissue culture polystyrene, TCPS) were used as control. After 1, 2, 3, 5 and 7 days of incubation, the adherent cells were analyzed by the MTT assay. Briefly, [3-(4, 5-cimethylthiazol-2-yl)-2, 5-diphenyl tetrazolium bromide] (MTT) (0.5 mg/ml) solution was added in each well and incubated for 3 h at 37°C. Dimethyl sulfoxide was then added to dissolve the crystals and the absorbance at 570 nm was measured by a microplate reader (SpectraMax M2*^e^*, Molecular Devices, USA).

#### 4.3 Measurement of intracellular reactive oxygen species (ROS)

MSCs (2×10^5^/well) were seeded in 6-well of FN or FN-Au coated culture plates for 24 hr. The generation of intracellular reactive oxygen species (ROS) was measured using the oxidation-sensitive fluorescent dye of 2′,7′-dichlorofluorescin diacetate (DCF-dA) (Sigma) [32]. Briefly, cells were washed twice on the second day after seeding with PBS buffer and incubated with 500 µl of PBS containing 20 µM of DCF-dA at 37°C incubator for 30 min. Cells were measured at 530 nm emission wavelength after excitation at 480 nm at 30-min intervals for up to 4 h using the FACS Calibur flow cytometer (Becton Dickinson, USA). An increase in fluorescence intensity represents the generation of net intracellular ROS. Fluorescein-positive cells were processed using the FCS software (Becton Dickinson, USA).

### 5. Protein expression of MSCs

#### 5.1 Gelatin zymography assay

Cells (5×10^5^ cells) were seeded in 10 cm culture dish with FN or FN-Au-coated and culture on condition medium for 48 h. After incubation, the conditioned medium was collected, centrifuged and assayed for gelatin zymography as previously described [13], [21]. Briefly, prepare gel was according to the standard procedure. Apply samples (typically 10–25 µl) and run the gel with 1× Tris-Glycine SDS runnung buffer according to the standard running conditions (∼125 V, constant voltage, 120 min). After running, gels were incubated in the Zymogram Renaturing buffer (100 ml for one or two mini-gels) with gentle agitation for 30 minutes at room temperature. After then, equilibrate the gel for 30 min at room temperature with agitation then replace with fresh 1× Zymogram developing buffer and incubate at 37°C overnight for maximum sensitively. Next, stained with 0.5% Coomassie brilliant blue R-250 (contain with 10% acetic acid and 45% methanol) and destained with 10% acetic acid (contain with 45% methanol). Areas of protease activity will appear as clear bands against a dark blue background where the protease has digested the substrate. MMPs gelatinase activity was then evaluated by quantitative densitometry. Data were normalized on the protein amount measured in cell supernatant. Images were process by Image Pro Plus 5.0 software (Media Cybernetics).

#### 5.2 Immunofluorescence analysis of CD68, eNOS, α5β3 and CD31 expression

Cells (2×10^4^ cells on each 15-mm FN or FN-Au-coated coverslips placed in the 24-well plate) were incubated in the condition medium as previously described. After incubation, the cells were fixed and permeabilized [13], [21]. After that, cells were incubated in the primary anti-CD68 antibody (1: 150 dilution, GeneTex InC, USA), primary anti-eNOS antibody (1: 300 dilution, Santa Cruz), anti-α5β3 integrin antibody (1: 300 dilution, Santa Cruz), and anti-CD31 antibody (1: 300 dilution, Santa Cruz). After further washes, cells were incubated with the appropriate secondary FITC-conjugated immunoglobulin (1: 300 dilution) (green color fluorescence) for 60 min. The cell nuclei were stained with DAPI (Invitrogen) (1 µg/ml) for 10 min. After washes, the samples were mounted on microscope slides with the storage solution (glycerol/PBS) and sealed with a synthetic mount.

#### 5.3 Flow cytometry analysis of αVβ3 integrin expression

Cells (5×10^5^ cells) were seeded in 10 cm culture dish with FN or FN-Au-coated culturte dish on the condition medium for 48 h. αVβ3 integrin expression level was detected by the flow cytometer. The expression of cellular αVβ3 integrin was analyzed as previously reported [13], [21]. After incubation, cells were collected by trypsinization and incubated with primary anti-αVβ3 integrin (10 µg/ml, Santa Cruz) for 1 h. Cells were then treated with secondary FITC immunoglobulin antibody and analyzed by the flow cytometer. Fluorescein-positive cells were processed using the FCS software (Becton Dickinson).

### 6. Migration ability of MSCs

FN or FN-Au coated coverslip glass were placed into each well of an Oris^(™)^ Cell Migration Assay Tri-Coated plate (Invitrogen). A cell seeding stopper (2 mm in diameter) was then placed in the central area on top of various coating. Oris^(™)^ seeding stoppers were seeded with ∼8000 MSCs (100 µl of 8×10^3^ cells/ml) and incubated for 24 h and 48 h incubation to reach confluency. The stopper was then removed from the test well, but remained in place in the pre-migration reference wells. The seeded plate was incubated in 37°C for observation of pre-migration (t = 0 h) and post-migration (24 to 48 h). Calcein AM (Sigma) (200 µl and 2 µM) containing serum free medium was added to each well and the image was captured by a fluorescence microscope (Zeiss Axio Imager A1, USA) after 24 and 48 h. The travelling distance of cells in the boundary was quantified by Image Pro Plus 5.0 software.

### 7. Statistical analysis

Multiple samples were collected in each experiment and expressed as mean ± standard deviation. All experiments were repeated independently to assure for reproducibility. Single-factor analysis of variance (ANOVA) method was used to assess the statistical significance of the results. *p* values less than 0.05 are considered significant.

## Results

### 1. Characterization of the FN-Au nanocomposites

**Figure 1:**
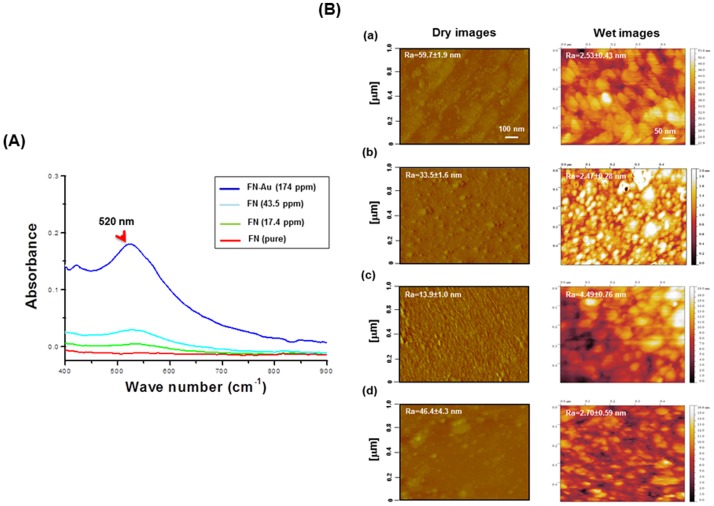
UV-Vis spectra analysis. (**A**) UV-Vis spectra for the pure FN and FN-Au nanocomposites containing 17.4 ppm, 43.5 ppm, and 174 ppm of AuNPs. (**B**) AFM topography diagrams for (a) the pure FN and FN-Au nanocomposites containing (b) 17.4 ppm, (c) 43.5 ppm, and (d) 174 ppm of AuNPs, measured in the dry or wet condition. All results are representative of one of three independent experiments. Ra is the average roughness of the sample.

The UV-Vis peak at 290 nm and 525 nm is shown in **Figure 1A**. The peak at 525 nm was attributed to the concentration increase of AuNPs from 17.4 ppm to 174 ppm. After coating, the surfaces in dry as well as in wet (culture medium) conditions were observed by AFM as shown **Figure 1B**. The dry surface of FN had an average roughness (Ra) of 59.7±1.9 nm, which was decreased to 33.5±1.6 nm in the presence of 17.4 ppm AuNPs. The surface morphology of FN-Au 43.5 ppm was more homogenous with an average roughness value of 13.9±1.0 nm. When the AuNP concentration was further increased to 174 ppm, the roughness significantly increased (46.4±4.3 nm). In the aqueous medium, FN surface appeared to be swollen with larger-size aggregates (∼70 nm) but lower average roughness (2.53±0.43 nm). The roughness did not change much for FN-Au 17.4 ppm and FN-Au 174 ppm (2.47±0.28 nm and 2.70±0.59 nm each in wet conditions). On the other hand, the roughness of wet FN-Au 43.5 ppm (4.49±0.76 nm) was significantly greater (p<0.05) than that of the other samples. In addition, the aggregates on FN-Au 43.5 ppm revealed a more spherical shape. By image analysis, the aggregates on the wet surface of FN-Au 43.5 ppm had an aspect ratio closer to one (1.2±0.1), which was significantly lower (p<0.05) than those observed in FN (1.9±0.2), FN-Au 17.4 ppm (1.5±0.1), and FN-Au 174 ppm (1.6±0.1).

**Figure 2:**
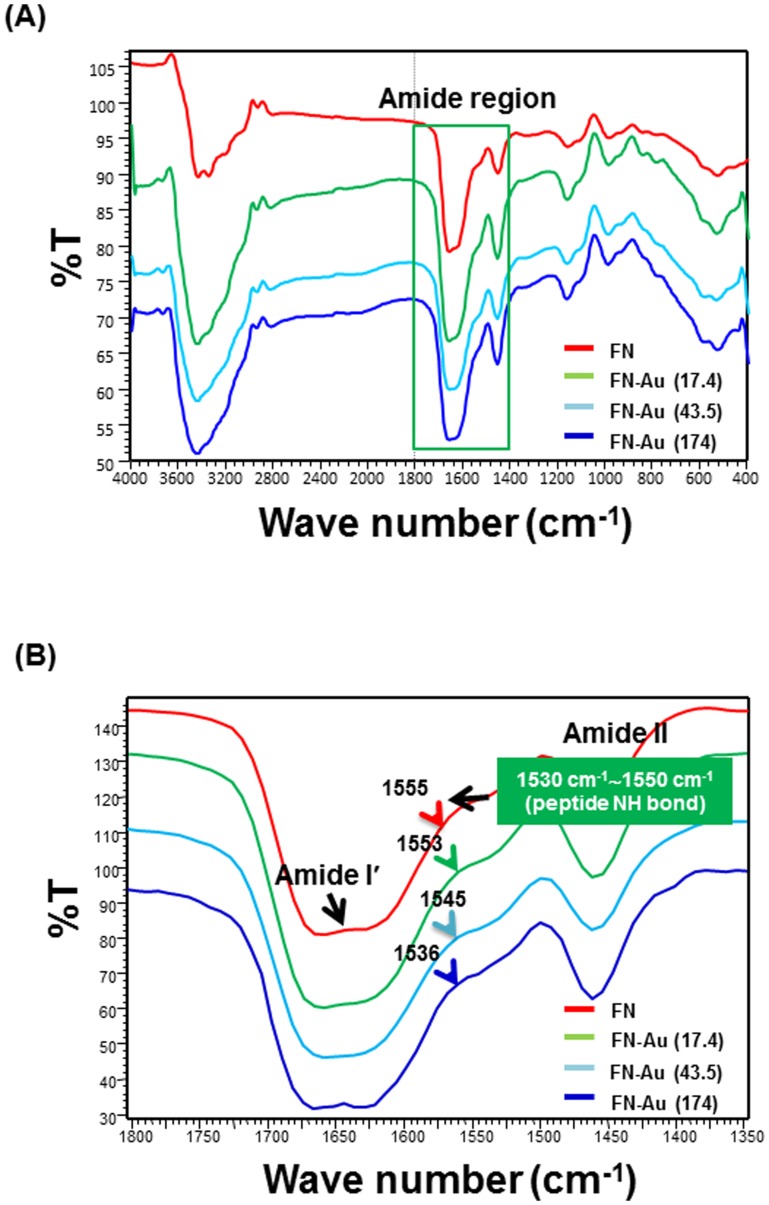
IR spectra of FN and FN-Au nanocomposites (A) in the total wavenumber range (400 cm^−1^ to 4000 cm^−1^) and (B) in the amide I′ (1650 cm^−1^∼ 1681 cm^−1^) region and amide II (1530 cm^−1^∼1550 cm^−1^) region. All results are representative of one of three independent experiments.

The IR spectrum of FN includes three major groups of bands, i.e. the amide II band, corresponding to the peptide NH bending vibration modes (1530∼1550 cm^−1^); the COO- antisymmetric stretching band, due to the Asp and Glu carboxylate groups (1565∼1585 cm^−1^); and the broad amide I' band, corresponding to the peptide carbonyl stretching modes ν (CO) (1610∼1700 cm^−1^) [33]. The incorporation of AuNPs into FN matrix caused a significant change in spectra near 1530∼1550 cm^−1^ (the peptide NH band). In **Figure 2**, there was a shift in the peak location of the peptide NH band from 1555 cm^−1^ for pure FN, to 1553 cm^−1^ for FN-Au 17.4 ppm, to 1545 cm^−1^ for FN-Au 43.5 ppm, and to 1536 cm^−1^ for FN-Au 174 ppm. This result suggested that the amide II may interact with AuNPs. In contrast, the change of band in amide I′ region was not as evident as that in the amide II region. FN is a dimeric adhesion molecule that consists of three types of repeating modules. Assembly of amino-terminal FN dimers can be incorporated into the extracellular matrix of fibroblasts [34]. Our results suggested that the peptide NH-bonding between FN and AuNPs may enhance cell-matrix interaction. This interaction may contribute in influencing the differentiation of stem cells.

### 2. Biocompatibility assay

**Figure 3:**
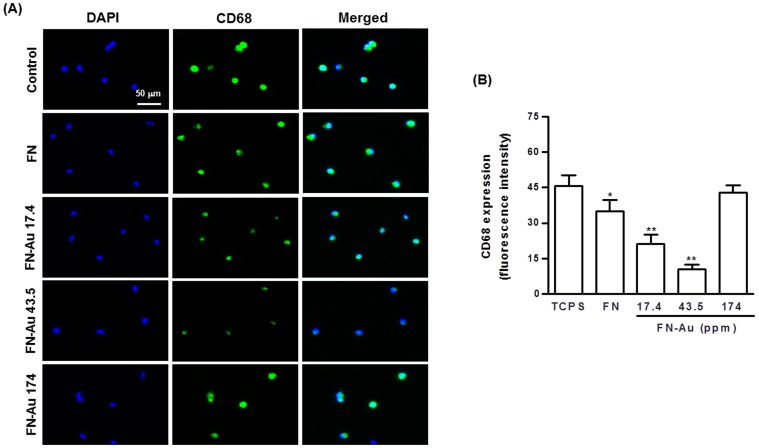
The expression of CD68 for macrophages on different materials at 96 h. (**A**) Cells were immunostained by the primary anti-CD68 antibody and conjugated with FITC-immunoglobulin secondary antibody (green color fluorescence) and cell nuclear staining was performed by DAPI (blue color staining). Results were taken by fluorescence microscopy. (**B**) CD68 expression was quantified based on fluorescence intensity. Data are mean ±SD (n = 3), *p<0.05, **p<0.01: smaller than control (TCPS).

Adherent monocytes progressed from the expected round monocyte morphology into the spread cytoplasm morphology of macrophages after 96 h of incubation on different materials, as shown in **Table 1**. FN-Au 43.5 ppm (9.62±2.11%) had a lower conversion rate, followed by FN-Au 17.4 ppm (17.50±3.76%), pure FN (21.56±5.41%), and FN-Au 174 ppm (24.70±3.92%). The activation effects on FN and on different FN-Au nanocomposites were all lower than that on the TCPS (27.01±5.11%). The monocyte activation on different materials was confirmed by CD68 staining of macrophages, as shown in **Figure 3A**. The semi-quantitative data of CD68 expression based on fluorescent intensity (**Figure 3B**) revealed a similar trend among different materials as that in **Table 1**. These results suggested that FN and FN-Au as the surface of a device may provoke a lower inflammatory response than TCPS.

**Table 1:** Human monocytes adhered and activated on control and test materials after 96 h culture.

Materials	The number of monocytes (×10^4^)	The number of macrophages (×10^4^)	Conversion (%)
Control (TCPS)	8.463±2.12	2.958±0.52	27.008±5.11
FN	8.618±2.37	2.275±0.81	21.565±5.41 *
FN-Au 17.4 ppm	9.091±1.79	1.186±0.33	17.501±3.76 **
FN-Au 43.5 ppm	9.322±1.28	0.825±0.11	9.622±2.11 **
FN-Au 174 ppm	8.525±1.69	2.683±0.81	24.704±3.92

* : p<0.05, greater than control (TCPS).

** : p<0.01, greater than control (TCPS).

**Figure 4:**
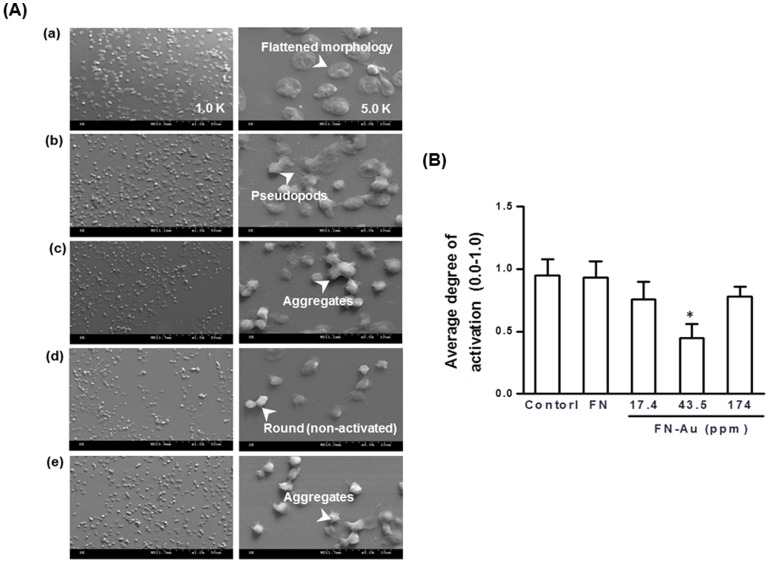
SEM images analysis. (A) SEM images showing the adhesion and activation of human blood platelets on the control group (glass), pure FN, and FN-Au nanocomposites containing 17.4 ppm, 43.5 ppm, and 174 ppm of AuNPs. (B) The average degree of platelet activation on different materials was quantified by image analysis. Data are mean ± SD (n = 3). *p<0.05: greater than control (TCPS). The activation of each platelet was scored (0.0–1.0) based on the morphological change.

The SEM images of platelets adhered on the surface of the pure FN and those containing 17.4, 43.5, and 174 ppm AuNPs are shown in **Figure 4A**. After 1 h incubation, the number of adhered platelets on control group (glass) was more than that on pure FN, and was followed by FN containing 17.4 ppm, 43.5 ppm, and 174 ppm of AuNPs. In addition, the platelets were aggregated and showed evident pseudopods on the control group (glass) and pure FN matrix. On the other hand, most platelets remained round morphology on the surface of FN-Au 43.5 ppm. The average extent of platelet activation on different materials (0.0–1.0) was ranked in the order of: control (glass) = FN>FN-Au 17.4 ppm = FN-Au 174 ppm>FN-Au 43.5 ppm (**Figure 4B**).

### 3. Cytoskeletal change of MSCs on FN-Au nanocomposites

**Figure 5:**
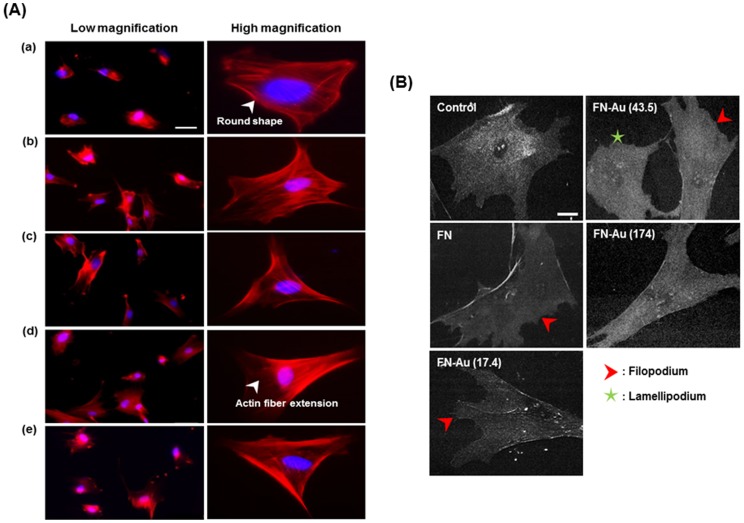
Cytoskeletal fibers examination. (**A**) Rhodamine phalloidin staining for the cytoskeletal fibers of MSCs on pure FN and different FN-Au nanocomposites at 8 h and 48 h observed by fluorescence microscopy: (a) control group (glass), (b) pure FN, (C) FN-Au 17.4 ppm, (d) FN-Au 43.5 ppm, and (e) FN-Au 174 ppm. Scale bar = 100 µm. (**B**) SEM images for MSCs on pure FN and different FN-Au nanocomposites at 48 h. (a) Control, (b) FN, (c) FN-Au 17.4 ppm, (d) FN-Au 43.5 ppm and (e) 174 ppm. Arrows indicate filopodia and stars indicate lamellipodia. Scale bar = 50 µm.

Observation of actin fiber extension by rhodamine-conjugated phalloidin staining is shown in **Figure 5A**. In the control group (glass), actin fibers exhibited the round shape and were mostly localized near the cell body. On FN-Au nanocomposites, actin fibers became more extended and dense, especially for cells on FN-Au 43.5 ppm. The quantitative data of cell area as represented in **Table 2**. SEM images in **Figure 5B** clearly showed that cells did not have protrusion on the control (glass) and the cell body was flattened. On the other hand, cells on FN-Au demonstrated more filopodia and lamellipodia, especially for those on PU-Au 43.5 ppm.

**Table 2:** Quantification data of cell area and size.

Materials	Area (µm^2^)	Size (length) (µm)	Size (width) (µm)
Control (Glass)	936.9±99.8	47.0±5.1	29.3±7.4
FN	1778.2±252.	72.0±5.5*	45.7±7.6
FN-Au 17.4 ppm	1650.9±227.5	88.0±3.7*	38.1±2.4
FN-Au 43.5 ppm	2211.8±374.9	136.6±20.4*	46.4±17.5
FN-Au 174 ppm	2344.7±229.9	73.5±1.3*	50.3±7.7

* : p<0.05: greater than control (Glass).

### 4. Cell growth and ROS generation

**Figure 6:**
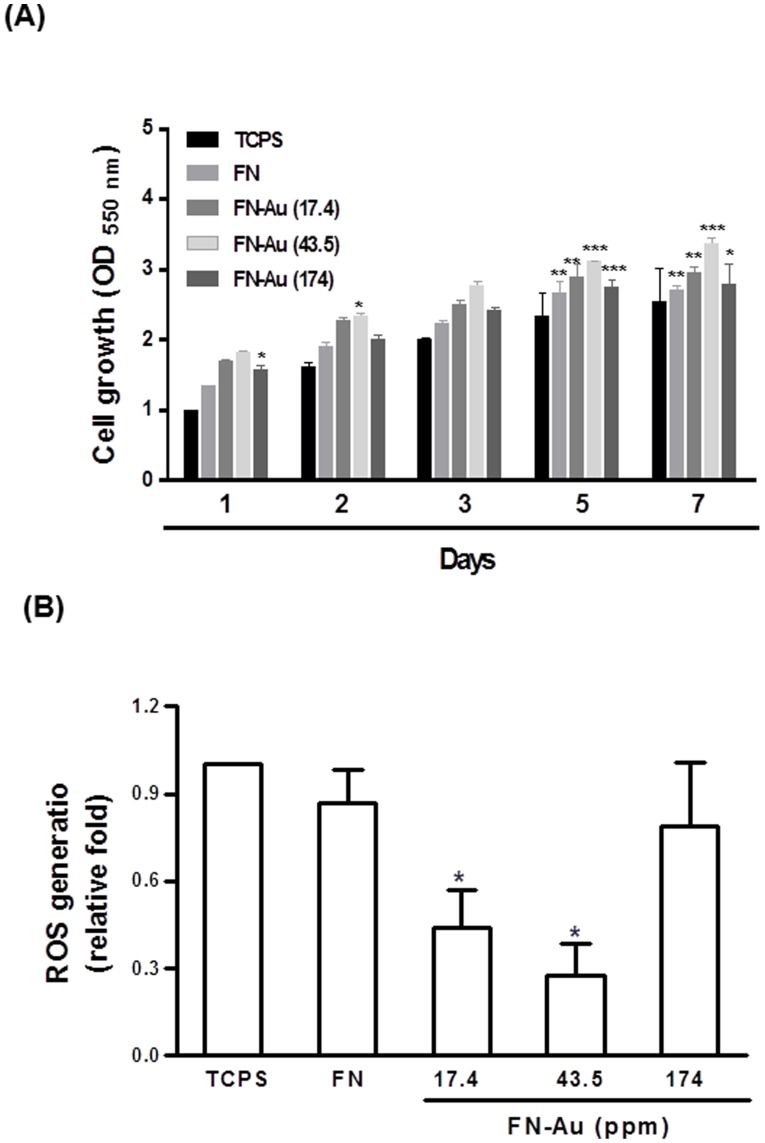
Cell proliferation assay. (**A**) MSC proliferation examined by MTT assay on control (TCPS), pure FN, and FN-Au nanocomposites containing 17.4 ppm, 43.5 ppm, and 174 ppm of AuNPs. Data are mean ± SD (n = 6). *p<0.05, **p<0.01, ***p<0.001: greater than control (TCPS). All results are representative of one of six independent experiments. (**B**) The intracellular reactive oxygen species (ROS) quantified by 2,7-dichlorofluorescein diacetate (DCFH-dA) and flow cytometric analysis for MSCs on different surfaces. Data are mean ± SD (n = 3). *p<0.05: greater than control (TCPS). All results are representative of one of three independent experiments.

MSC proliferation on various materials is shown in **Figure 6A**. Cells proliferated better on all FN-Au nanocomposites than that on TCPS after 1, 2, 3, 5, and 7 days of incubation. In particular, FN-Au 43.5 ppm showed the most cell growth even after 7 days. Proliferation of MSCs on different materials was ranked in the order of FN-Au 43.5 ppm>FN-Au 17.4 ppm>FN-Au 174 ppm>pure FN>TCPS. ROS generation of MSCs on various materials is depicted in **Figure 6B**. FN-Au nanocomposites at all concentrations (17.4–174 ppm) induced a lower amount of ROS generation, in particular for FN-Au 43.5 pm. ROS generation on different materials was ranked in the order of TCPS ∼ FN ∼ FN-Au 174 ppm>FN-Au 17.4 ppm>FN-Au 43.5 ppm.

### 5. The effect of FN-Au nanocomposites on the expression of αVβ3 integrin, eNOS and the activity of MMP

**Figure 7:**
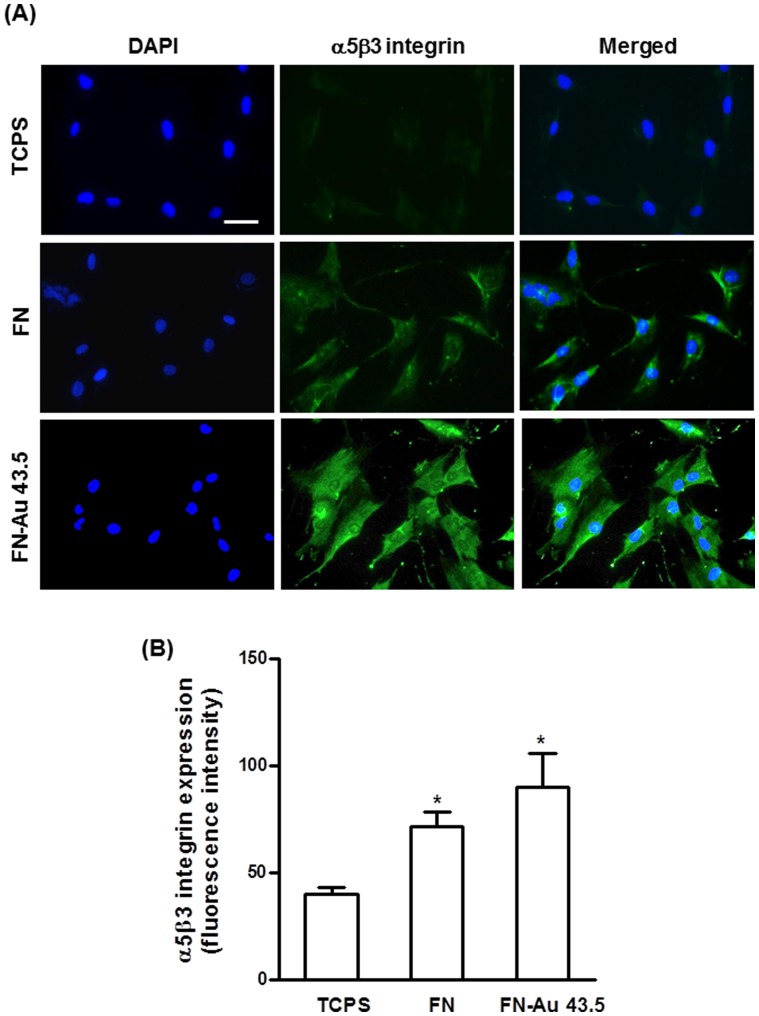
The expression of αVβ3 integrin of MSCs on different materials at 48 h. (**A**) MSCs were immunostained by the primary anti-αVβ3 integrin antibody and conjugated with FITC-immunoglobulin secondary antibody (green color fluorescence) and cell nuclear staining was performed by DAPI (blue color staining). Results were taken by fluorescence microscopy. (**B**) The expression intensity of αVβ3 integrin in MSCs culture on different materials for 48 h quantified by flow cytometry. Data are mean ± SD (n = 3). * p<0.05: greater than control (TCPS).

Activation of αVβ3 integrins by FN-Au nanocomposites was observed. As shown in **Figure 7A**, FN-Au 43.5 ppm enhanced the most αVβ3 integrin expression. FN also induced αVβ3 integrin expression. Meanwhile, the expression level of αVβ3 integrin quantified by flow cytometry analysis also followed a similar pattern (**Figure 7B**). Here, in this study the expression level of αvβ3 integrin on FN-Au 43.5 ppm was significantly higher than that of α5β1 integrin expression (**Figure S2**).

**Figure 8:**
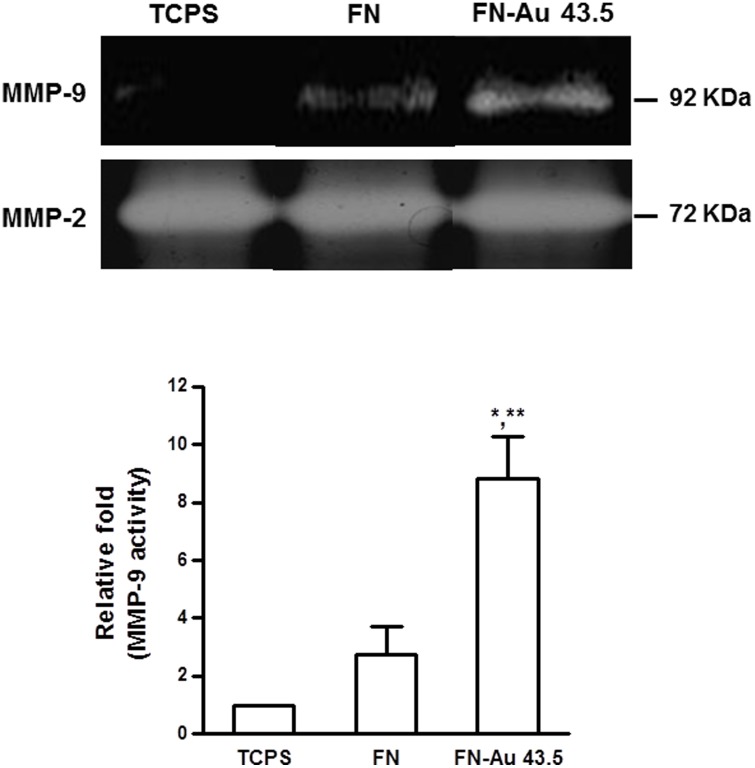
The MMP enzymatic activities for MSCs on FN-Au 43.5 ppm at 48 hr normalized to the protein content was evaluated and compared with that on control (TCPS) and pure FN. A representative zymogram for MMP-2 and MMP-9 is shown. Semiquantitative measurement of the optical density (OD) of gelatinolytic bands revealed significantly greater MMP-9 expression for MSCs on FN-Au 43.5 ppm. Data are the mean ± SD (n = 3). *p<0.05: greater than control (TCPS), **p<0.05: greater than FN.

**Figure 9:**
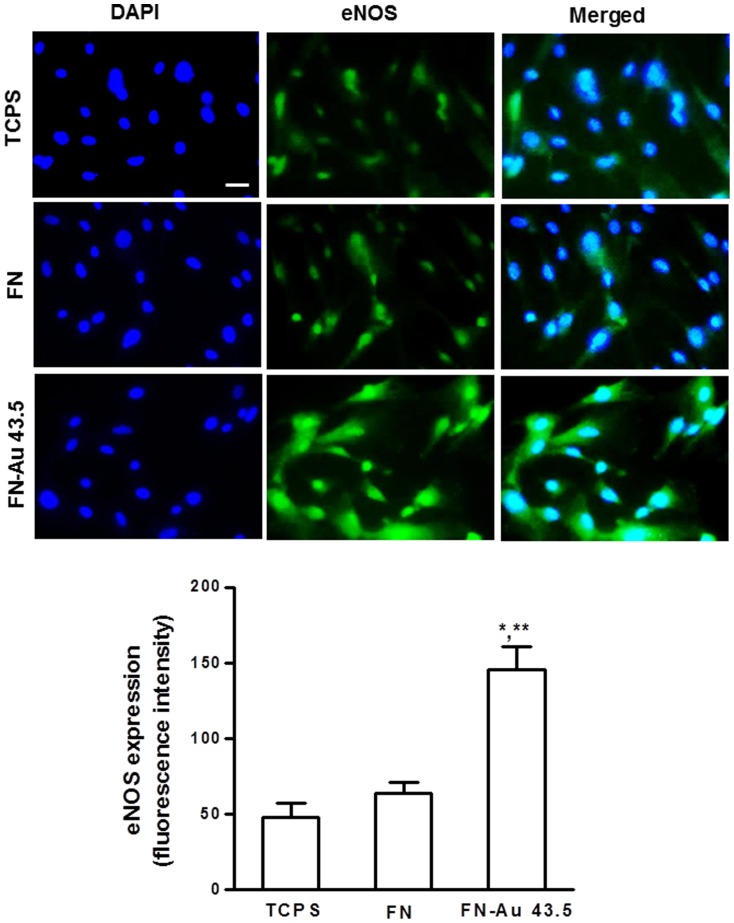
Expression of eNOS protein for MSCs examined by fluorescence microscopy. Cells were cultured on control group (TCPS), pure FN, and FN-Au 43.5 ppm for 48 h and stained with primary anti-eNOS antibody followed by FITC-conjugated immunoglobulin (green color fluorescence); cell nuclear staining was performed by DAPI (blue color staining). Scale bar = 10 µm. Semiquantitative measurement of immunofluorescence expression intensity revealed significantly higher level of eNOS expression compare with the control. Data are mean ± SD (n = 3). * p<0.05: greater than control (TCPS), ** p<0.05: greater than FN.

The expression of both MMP-2 and MMP-9 on MSCs was found to be regulated by FN-Au nanocomposites. As shown in **Figure 8**, the increase in the MMP-9 expression level on FN-Au vs. FN or TCPS was remarkable. On the other hand, the expression of MMP-2 was not significantly induced by FN-Au. The expression of MMP-9 on FN-Au was about three-fold higher than that on the pure FN. The expression of eNOS protein at 48 h of incubation was observed by immunofluorescence staining, as illustrated in **Figure 9**. The higher eNOS fluorescence intensity in the cytoplasma for MSCs on PU-Au 43.5 ppm was about three-fold higher than those on pure FN and TCPS.

### 6. The migration ability of MSCs on FN-Au nanocomposites

**Figure 10:**
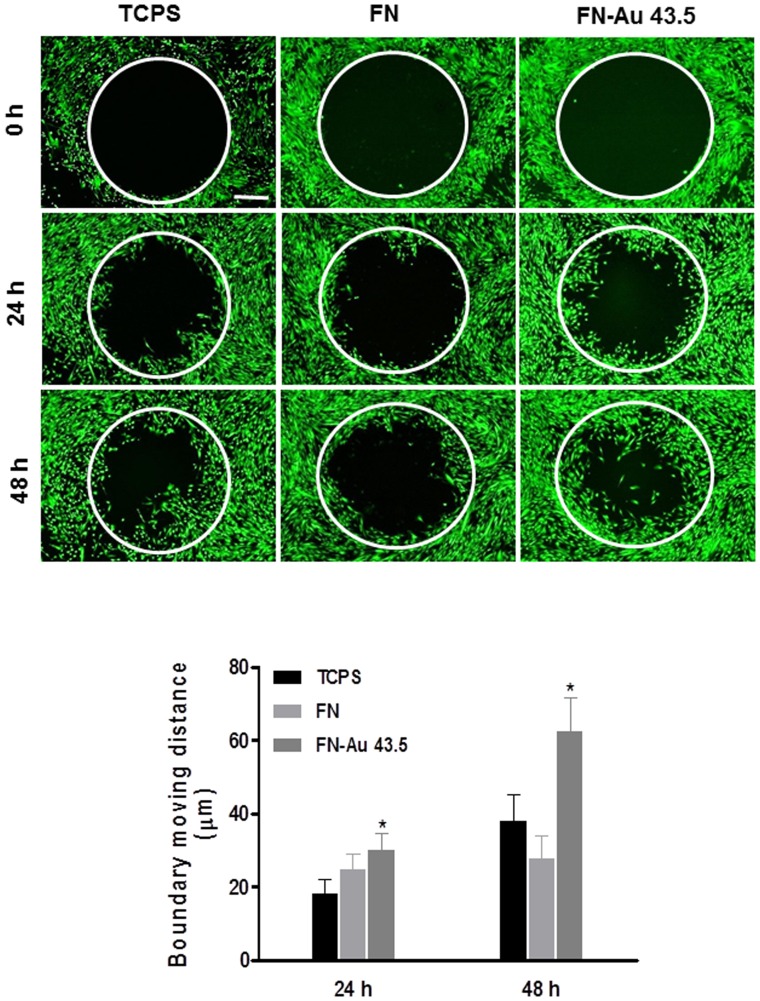
The migration ability for MSCs on control (TCPS), pure FN, and FN-Au 43.5 ppm. Cell migration into the gap zone area was monitored by fluorescence microscopy. After 24 h and 48 h of incubation, cells were stained by calcein-AM (2 µM) prior to examination. Data are the mean ± SD (n = 3). *p<0.05: greater than control (TCPS).

The real-time images of cell boundary captured in pre-migration (t = 0 h) and post-migration (t = 24 and 48 h) wells are shown in **Figure 10**. The traveling distance of boundary cells may be affected by the cell growth rate in each group. However, since the cells were nearly confluent after 24 h, the boundary moving distance may to some extent reflect cell migration ability. It was found that the average moving distance during 0–24 h on FN-Au 43.5 ppm (30.03±4.27 µm) was significantly greater than those on pure FN (24.9±4.23 µm) and TCPS (18.2±3.9 µm). The average moving distance during 24–48 h on FN-Au 43.5 ppm (62.4±9.21 µm) was also significantly greater than those on pure FN (37.95±6.21 µm) and TCPS (27.95±7.22 µm).

### 7. CD31 expression of MSCs on FN-Au nanocomposites

**Figure 11:**
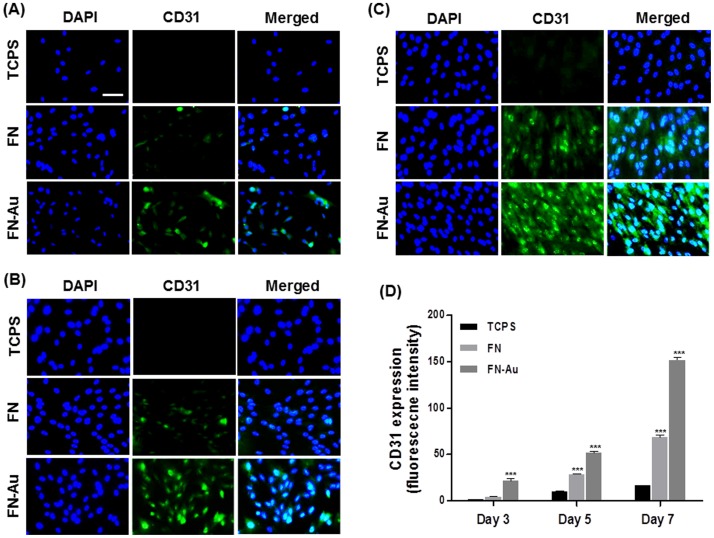
The CD31 protein expression of MSCs on different materials at 3, 5, and 7 days. (**A**) MSCs were immunostained by the primary anti-CD31 antibody and conjugated with FITC-immunoglobulin secondary antibody (green color fluorescence) and cell nuclear staining was performed by DAPI (blue color staining). Results were taken by fluorescence microscopy. Scale bar = 10 µm. (**B**) Semiquantitative measurement of fluorescence intensity revealed significantly higher level of CD31 expression compare with the control (TCPS). Data are mean ± SD (n = 3). *** p<0.01: greater than control (TCPS).

The real-time PCR analysis showed that the EC marker genes (vWF and CD31) were significantly upregulated after MSCs were cultured on FN-Au 43.5 ppm for 5 days (**Figure S3**). We then carried out the immunofluorescence staining analysis to characterize the CD31 expression of cells (EC phenotype) at 3, 5, and 7 days. The results are shown in **Figure 11**. After 3 days of incubation, the CD31 expression was observed for cells on FN-Au 43.5 ppm but was very low for cells on TCPS and the pure FN. After 5 days and 7 days of incubation, the CD31 expression was significantly increased in all groups, especially for cells on FN-Au, which strongly suggested that FN-Au nanocomposite films may promote MSCs differentiation into mature ECs.

## Discussion

Biocompatibility is the most important aspect of a biomaterial intended for implantation in the human body. Different surface modification methods, including chemical and physical modification and biomolecule immobilization, have been used to improve the biocompatibility. One of the methods is to change the surface topography on the micron and nanometer scale, which may lead to changes in surface chemistry as physicochemical cues are intrinsically linked. This modification may encourage desirable protein, cellular, and tissue interactions at the cell-biomaterial or blood-biomaterial interface, thus improving the performance of the biomaterial. Surface roughening of a range of materials including polymers have been shown to enhance cellular adhesion [35]–[40]. Some of these proteins are selective such as fibronectin where they mediate vascular cell interactions with the polymer [37].

This study attempted to employ a functionalized surface coating made of AuNPs and FN to improve the biocompatibility as well as the bioactivity that could have favorable cellular response. The surface morphology of the FN coating was significantly altered by the existence of a small amount of AuNPs. FTIR results showed that there was interaction between AuNPs and the amide II region (peptide NH bond) of the pure FN matrix on the material surface. These results indicated that the chemical property of FN was also changed by the presence of AuNPs.

FN-Au nanocomposites at 17.4–174 ppm of AuNPs all showed better biocompatibility than the pristine FN coating. These include reductions in both monocyte activation and platelet activation. In another word, AuNPs appeared to provide a reasonable means to attenuate the inflammatory response or unwanted reaction (e.g. thrombus formation) to biomaterials. Antioxidation was associated with reduced host inflammation after implantation of biomaterials [41], [42]. Implantation of antioxidant-modified polymer into rats reduced the PMN-rich acute inflammatory infiltrates [43]. Therefore, the antioxidant effect of AuNPs may account for the favorable changes in FN-Au nanocomposites compared with FN.

In addition to the antioxidant effect, the surface properties of FN matrix could be modified by applying a small amount of AuNPs of the optimal size and concentrations. Literature has reported the effect of nanometric features on the cellular response. For example, surface nanoislands (13–27 nm) were indicated to promote the response of fibroblasts [39]. Poly(ε-caprolactone)/poly(ethylene glycol) diblock copolymer with 27 nm high islands obtained by phase separation had better biocompatibility [40]. In the present study, FN-Au 43.5 ppm with surface roughness ∼14 nm exhibited better cellular response than the pure FN and the other FN-Au nanocomposites. These include cell adhesion, proliferation, as well as migration effects. Besides, FN-Au could reduce the amount of ROS generation and the inflammatory response of MSCs. This may be due to the ROS scavenging ability of FN-Au or elimination of free radicals by AuNPs on the surface of FN, which requires further verification.

Optimal cell attachment, migration, proliferation, and differentiation on a biomaterial are essential for the wound healing effect of MSCs. The secretion of MMPs is involved in enhancing cell migration and vascular remodeling [44]. Proteinases such as MMP-2 and MMP-9 contribute to cell migration through its ability to activate cell surface molecules such as αvβ3 integrin [45]. Stromal-derived factor (SDF-1) also stimulates the expression of specific cell surface markers (e.g. α5β3 integrin), which promotes stem cell homing to the site of vascular damage. Our data showed that the αvβ3 integrin as well as MMP-9 expression was induced by FN-Au 43.5 ppm. These observations may be associated with the increased EC phenotype of MSCs.

The cardiovascular protective role of the endothelium is associated with NO [46] produced by eNOS, which activates EC migration [47]. MSCs play critical functions in normal development and wound repair. MSCs may not only differentiate into ECs [48], but also exert paracrine effects, which enhance the repairing capacity of implanted MSCs by increasing their migration [49]. In this study, FN-Au 43.5 ppm activated a greater level of eNOS expression and faster migration of MSCs than the other FN-Au nanocomposites and pure FN. We also observed that FN-Au 43.5 ppm may promote the EC differentiation of MSCs. The increased EC phenotype of MSCs on FN-Au 43.5 ppm may give rise to greater tissue repair.

Based on our current study, FN with 43.5 ppm of AuNPs showed better biological performances, including enhanced cell proliferation, reduced platelet and monocyte activation, and lower ROS generation than the original FN, followed by FN-Au 17.4 ppm and FN-Au 174 ppm. The findings suggested a modification of cellular responses by the nanocomposites with a moderate loading of AuNPs. Previously we have demonstrated that an overloading of AuNPs can lead to particle aggregation in the polymer matrix [11], [12], and in such a case the beneficial effect can decrease significantly. As observed in this study, FN-Au 43.5 ppm was smooth, which may be associated with the moderate loading and the optimal interaction between FN and AuNPs. The smooth surface was not seen at higher AuNPs (174 ppm) in dry state, possibly because of aggregation of AuNPs. The performance of FN-Au was thus closely associated with how well the AuNPs were dispersed and interacted with FN matrix. On the other hand, the roughness significantly decreased in wet conditions for all samples. Interestingly, FN-Au 43.5 ppm had the lowest roughness in the dry state but the highest roughness in the wet state. In addition, wet FN-Au 43.5 ppm consisted of large (∼70 nm) spherical particles, which were suspected to be highly swollen aggregates. It could be that the well hydration of FN-Au 43.5 ppm may keep FN more native. The more detailed effect of AuNP contents on the structure and biological activity of the FN-Au nanocomposites will be a future subject of study.

FN-Au nanocomposite coating simultaneously improved the blood compatibility and MSC response. It is anticipated that these FN-Au nanocomposite coatings would have a potential for improving the biocompatibility of clinical blood-contacting devices. Besides, the promoted migration and EC phenotype of MSCs may increase the wound healing through the integrin/MMP signaling.

## Conclusion

The effects of FN and it composites with AuNPs on the biocompatibility and MSC response to the coated surface were investigated. FN-Au coating containing 43.5 ppm of AuNPs significantly reduced the reactions of monocytes and platelets as well as altered the behavior of MSCs. The physicochemical changes of FN-Au may play a crucial role in modulating the cellular response to the FN nanocomposites. This study provides a simple method to increase the bioactivity of natural biomolecules (such as FN) by mixing them with a proper amount of AuNPs. The nanocomposite coating (such as FN-Au) may be a potential surface coating for implanted cardiovascular devices.

## Supporting Information

Figure S1 **Characterization of MSCs.** (a) Morphology of MSCs examined by optical microscopy. (b) MSC specific marker expression by flow cytometry. MSCs were stained with PE or FITC-conjugated antibodies against the indicated markers: CD4, CD29, CD34, CD44, CD45, CD73, CD90, and CD105. An antibody isotype was used as control group. Data are mean ± SD.
(TIF)

Figure S2 **The expression of α5β1 integrin of MSCs on different materials at 48 h.** (**A**) MSCs were immunostained by the primary anti-α5β1 integrin antibody and conjugated with FITC-immunoglobulin secondary antibody (green color fluorescence) and cell nuclear staining was performed by DAPI (blue color staining). Results were taken by fluorescence microscopy. (**B**) The intensity of α5β1 integrin expression in MSCs cultured on different materials for 48 h quantified by flow cytometry. Data are mean ± SD (n = 3). * p<0.05: greater than control (TCPS).
(TIF)

Figure S3 **The real-time RT-PCR analysis for the mRNA expression levels of EC markers (vWF and CD31) in MSCs after culture for 5 days on pure FN and FN-Au nanocomposites containing 17.4, 43.5, and 174 ppm of AuNPs.** GAPDH was used as the internal control. The results were presented as the ratio of EC marker to GAPDH signals for each condition, normalized to control.
(TIF)
