# Supplementary figures and images for: Biocompatibility and Favorable Response of Mesenchymal Stem Cells on Fibronectin-Gold Nanocomposites
Source: PLoS One. 2013 Jun 24;8(6):e65738. doi: 10.1371/journal.pone.0065738 (PMC3691216; doi:10.1371/journal.pone.0065738)

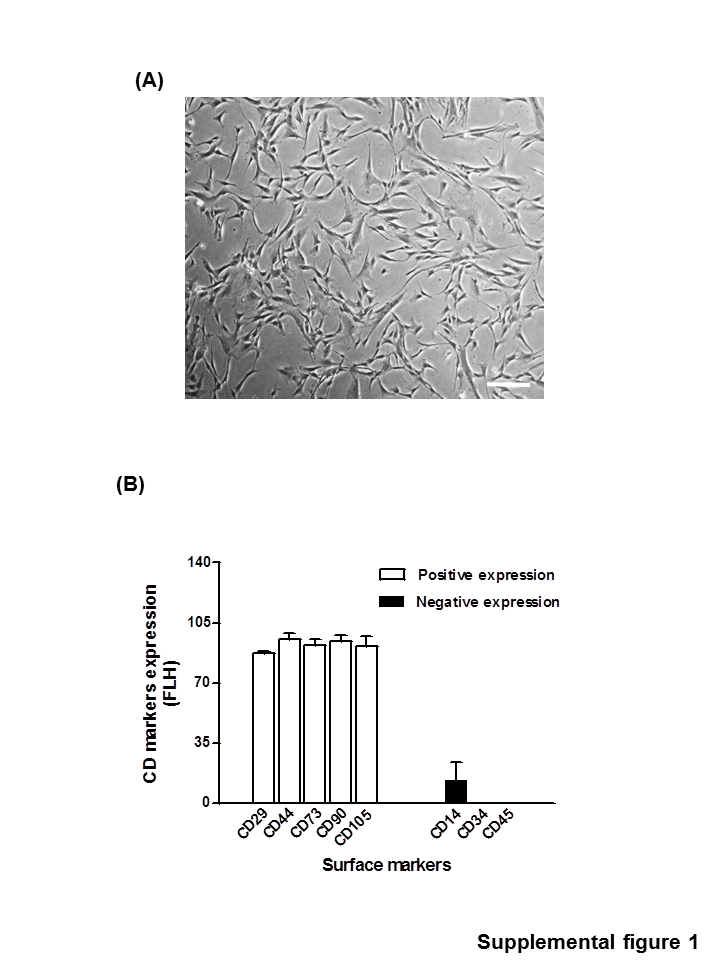

Supplement: Figure S1 — Characterization of MSCs. (a) Morphology of MSCs examined by optical microscopy. (b) MSC specific marker expression by flow cytometry. MSCs were stained with PE or FITC-conjugated antibodies against the indicated markers: CD4, CD29, CD34, CD44, CD45, CD73, CD90, and CD105. An antibody isotype was used as control group. Data are mean ± SD. (TIF) [file pone.0065738.s001.tif]

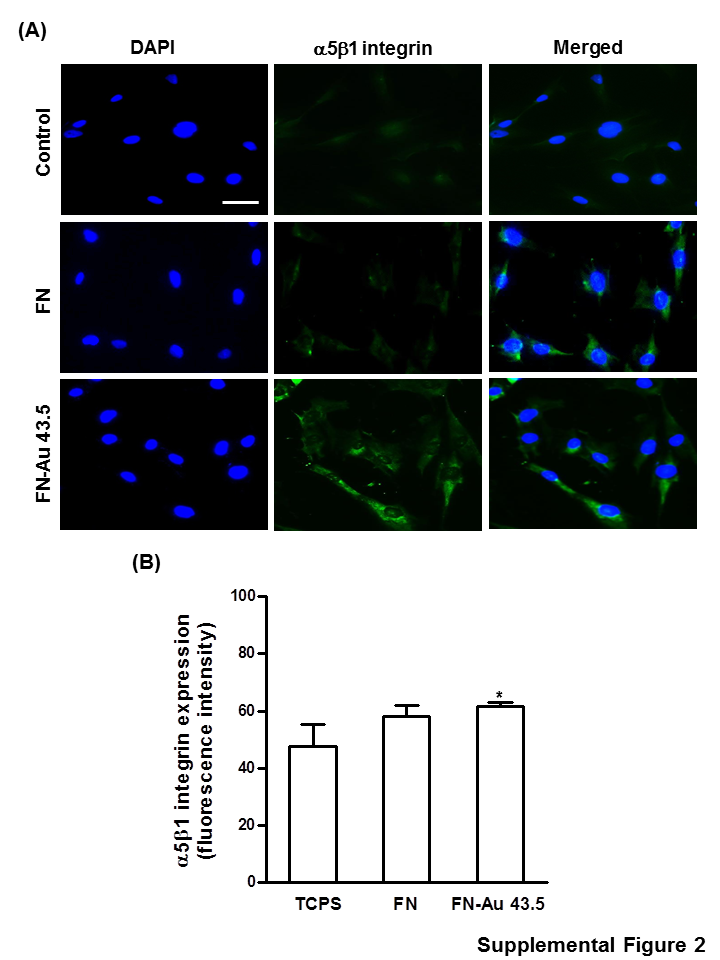

Supplement: Figure S2 — The expression of α5β1 integrin of MSCs on different materials at 48 h. (A) MSCs were immunostained by the primary anti-α5β1 integrin antibody and conjugated with FITC-immunoglobulin secondary antibody (green color fluorescence) and cell nuclear staining was performed by DAPI (blue color staining). Results were taken by fluorescence microscopy. (B) The intensity of α5β1 integrin expression in MSCs cultured on different materials for 48 h quantified by flow cytometry. Data are mean ± SD (n = 3). * p<0.05: greater than control (TCPS). (TIF) [file pone.0065738.s002.tif]

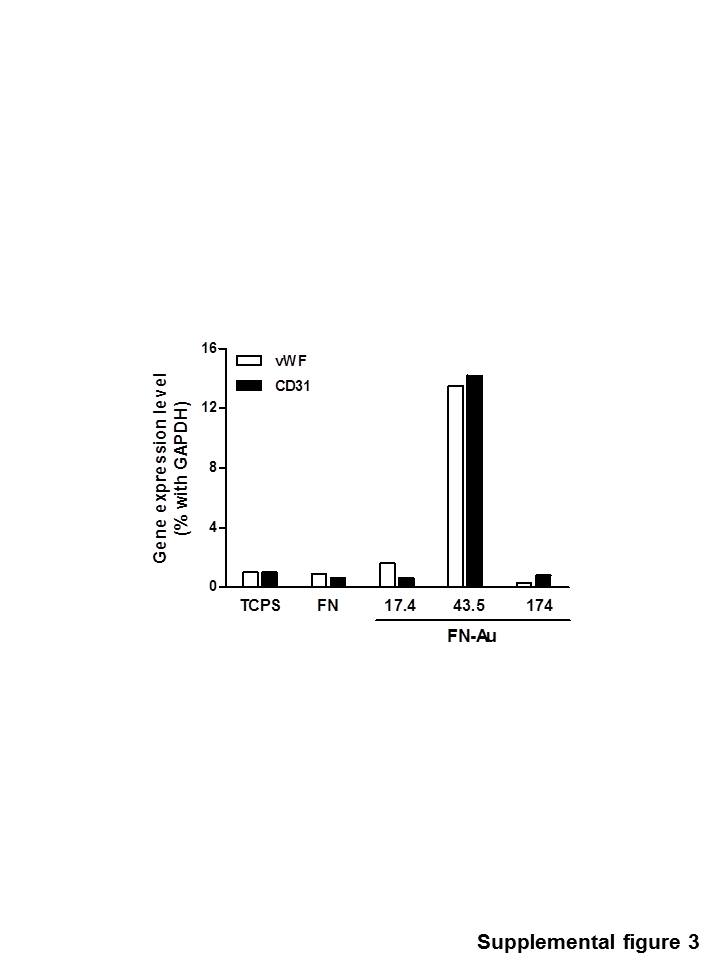

Supplement: Figure S3 — The real-time RT-PCR analysis for the mRNA expression levels of EC markers (vWF and CD31) in MSCs after culture for 5 days on pure FN and FN-Au nanocomposites containing 17.4, 43.5, and 174 ppm of AuNPs. GAPDH was used as the internal control. The results were presented as the ratio of EC marker to GAPDH signals for each condition, normalized to control. (TIF) [file pone.0065738.s003.tif]
